# Surface Characterization and Optical Properties of Reinforced Dental Glass-Ceramics Related to Artificial Aging

**DOI:** 10.3390/molecules25153407

**Published:** 2020-07-28

**Authors:** Liliana Porojan, Roxana-Diana Vasiliu, Mihaela-Ionela Bîrdeanu, Sorin-Daniel Porojan

**Affiliations:** 1 Department of Dental Prostheses Technology (Dental Technology), “Victor Babeș” University of Medicine and Pharmacy Timișoara, Romania, Eftimie Murgu Sq. no. 2, 300041 Timișoara, Romania; sliliana@umft.ro; 2 National Institute for Research and Development in Electrochemistry and Condensed Matter, 300569 Timisoara, Romania; mihaelabirdeanu@gmail.com; 3 Department of Oral Rehabilitation (Dental Technology), “Victor Babeș” University of Medicine and Pharmacy Timișoara, Romania, Eftimie Murgu Sq. no. 2, 300041 Timișoara, Romania; porojan.sorin@umft.ro

**Keywords:** glass-ceramic, thermal cycling, optical properties, roughness, surface topography

## Abstract

The development of various dental glass-ceramic materials and the evolution of novel processing technologies lead to an essential change in the clinical and technical workflow. The long-term success of a dental restoration treatment is defined by its durability, which is directly influenced by the oral environment. This study’s purpose was to evaluate the artificial aging behavior of nanostructured, respective microstructured ceramics related to surface topography, roughness, and optical properties. Six monolithic restoration materials were selected: milled lithium disilicate glass-ceramic (LDS-M) MT (medium translucency), hot-pressed lithium disilicate glass-ceramic (LDS-P) MT and HT (high translucency), milled zirconia-reinforced lithium silicate ceramic (ZLS-M) MT and hot-pressed zirconia-reinforced lithium silicate ceramic (ZLS-P) MT and HT, resulting *n* = 96 surfaces. All the samples were artificially aged by thermal cycling, and all investigations were made before and after thermal cycling. In terms of optical properties, differences recorded between ZLS and LDS ceramics are not significant. Thermal cycling increases the translucency of ZLS and LDS glass-ceramic materials significantly, with the most harmful effect on the pressed and polished samples. Micro- and nano roughness are significantly influenced by in vitro aging and a negative correlation was recorded. Glazed samples are characterized by significant rougher surfaces for all types of materials. On nanolevel, ZLS materials are significantly smoothed by thermal cycling.

## 1. Introduction

Various glass-ceramic materials have been promoted and introduced in dentistry, associated with the evolution of novel processing technologies. These lead to an essential change in the clinical and technical workflow, along with the changes in treating patients. The trend towards achieving monolithic restorations is related to the chipping or delamination behavior of multilayered aesthetic restorations [1].

The term glass-ceramics is defined by one glassy amorphous phase into which crystals are precipitated in a controlled manner by nucleation and crystal growth. The controlled precipitation of the crystalline phases (by temperature and time) permits us to overcome some of the glass deficiencies [2,3]. Controlled crystallization of glasses is known to positively impact dental ceramic materials mechanical properties [2,4].

Nanostructures can be adapted to confer excellent properties, related both to the surface and their inner structure. It is well known that the surface roughness has a substantial influence on the clinical behavior of the restorations [2]. Roughness increases the potential surface area for a material–environment interaction.

Glass-ceramics can be used from veneers to full anatomic restorations, according to ISO 6872. The materials requirements specified in this standard, associated with aesthetics, are decisive for the selection of the material. Due to the high demand for monolithic aesthetic restorations, high strength materials have been developed in this category, having appropriate optical properties [5,6,7,8].

Lithium disilicate glass-ceramics are extensively used in practice. In this class of materials, the interlocking microstructure together with a high content of crystalline phase (60–70 vol.%), formed by the lath-like Li_2_Si_2_O_5_ crystals, is the basis for the high strength (400–610 MPa) and toughness (2.3–2.9 MPa) of the material [5,9]. The crystallization of the lithium disilicate is controlled by a heating cycle, in which lithium metasilicate (Li_2_SiO_3_) reacts with the glassy phase (SiO_2_) to originate lithium disilicate (L_i2_Si_2_O_5_). The excellent mechanical properties are a result of decreasing in the size of the platelet-shaped crystals (length of 2000–3000 nm) and of increasing in interlocking among crystals [1,10,11,12].

The subsequent evolution of glass-ceramics led to the development of glass-ceramics reinforced with polycrystalline ceramics. These new glass-ceramics contain lithium silicate as the main crystalline phase in a vitreous matrix reinforced with zirconium dioxide crystals (~10%). The lithium silicate crystals have a size from 500 up to 1000 nm and up to 6 times smaller than lithium disilicate crystals present in lithium disilicate glass-ceramics. Zirconia particles act as an additive, influencing the crystallization by hindering crystal growth. The inner structure containing smaller crystals leads to excellent mechanical and optical properties and induces good surface finishing [1,13,14,15].

Optical properties of the glass-ceramics, such as translucency and opalescence, are critical factors for aesthetics and the natural appearance of the ceramic restorations. Several factors, such as the ceramic thickness, processing technique, composition, inner structure, crystalline content, grain size, pores, additives, and surface roughness, and topography, can influence the optical properties [16,17,18,19]. Surface finishing of ceramic materials is generally glazing, but mechanical polishing without a firing process can also be recommended for new types of micro- and nanostructured ceramics. After finishing, the surface becomes smoother, with benefits for the ceramic biocompatibility, decreasing the plaque accumulation and antagonist tooth wearing, and the susceptibility to staining, with a benefit for the natural-looking long-term aesthetics of the restoration [16,20,21].

Translucency (TP) is defined as the difference in color at a uniform thickness measured by a spectrophotometer device over white and black backings. If the material is completely opaque, the TP value is zero. As the TP value increases, the translucency of the material also increases. Opalescence is defined as a scattering of wavelengths of the visible light, and as a result, an object appears bluish, and an orange/brown in the reflected and transmitted color [17,18,21,22,23,24].

Also, the behavior of the aesthetic dental restorations during their clinical use is essential. In vitro studies may simulate the oral environment to study the behavior of the restorations, based on the type of material and processing method, to achieve reliable restorations. The clinical success of the dental restoration is defined by its longevity, which is substantially influenced by the oral environment. For a reasonable estimation of the long-term stability of dental restorative materials in the clinical situation, artificial aging tests to simulate oral environmental variables, and thermal cycling should be designed [25,26,27].

The thermal aging of dental materials is a natural phenomenon and is unavoidable. Therefore each ceramic material we use for dental restorations will age, and this aging will influence their clinical performance. Dissolution during clinical use is essential for glass-ceramic materials, as they predominantly made of silica glass structure with various other cations incorporated, which disrupt the silica network.

For the glass-ceramic materials that contain crystalline components, the difference in the dissolution rate may lead to increased surface roughness. Factors contributing to the aqueous dissolution of silicate-based dental ceramics and induced destabilization of Y-TZP zirconia are included in low-temperature degradation. Glass-ceramic materials will undergo relatively low dissolution rates with the rate and will generally develop a slightly hydrated porous layer depleted of the soluble species and will be softer than the underlying glass. The presence of crystalline species within the glass is likely to show the differential dissolution of the glass versus the crystalline phase. For lithium disilicate containing glass-ceramics, the crystalline Li_2_SiO_3_ phase appears to dissolve more slowly than the glass matrix resulting in the development of a rough surface [20,21,28].

The oral environment contributes to long-time degradation. New ceramic materials with different microstructure draw the attention from this point of view because a material that is the most strength is not necessary to respond better to aging and have a higher survival rate [29,30,31]. The question is which type of processing and finishing technologies should be used for each type of ceramic to achieve reliable aesthetic dental restorations. Modification of the surface could be a way for the mitigation of aging [32,33,34].

This study aimed to evaluate the artificial aging behavior of nanostructured, respective microstructured ceramics related to surface topography, roughness, and optical properties, to evaluate the long-term reliability of aesthetic monolithic dental restorations.

## 2. Results

### 2.1. Surface Roughness

**Figure 1:**
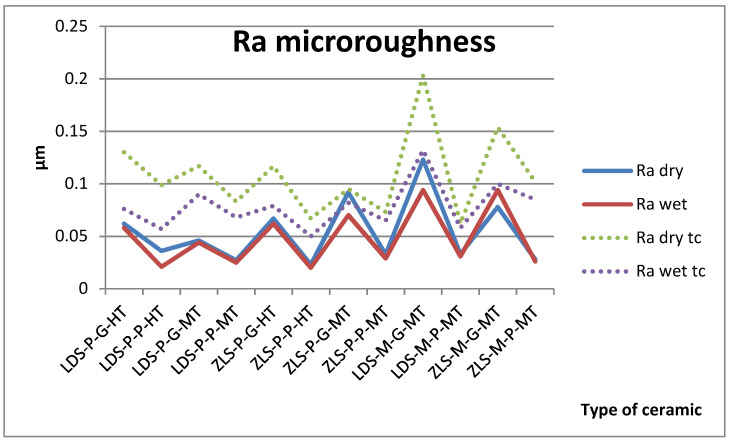
Arithmetic average roughness (Ra) microroughness values for all tested samples, dry and wet, before and after thermal cycling (tc).

**Figure 2:**
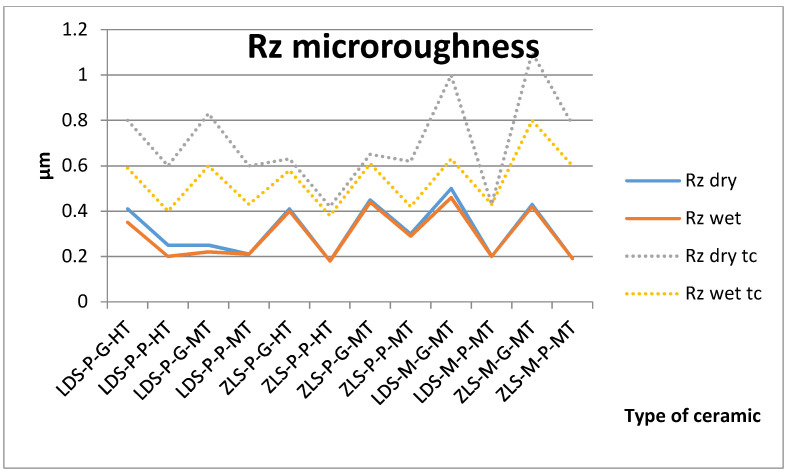
Absolute vertical roughness (Rz) microroughness values for all tested samples, dry and wet, before and after thermal cycling (tc).

The measurements were made in the same point. Surface roughness measurement results of Ra and Rz are described in Figure 1 and Figure 2.

The *t*-test demonstrated that the type of ceramic, processing procedures, the wetting of the surface do not have a significant impact on the microroughness on the samples, only surface processing (polishing or glazing) had a significant impact, before thermocycling. Glazed surfaces have significantly higher roughness than polished ones.

For the aged samples, significant differences are registered between dry and wet surfaces and between glazing and polishing (Table 1). After thermocycling, glazed surfaces have significantly higher values than polished and dry than wet. All samples are influenced by thermal cycling; the roughness increases.

Ra values are under 0.12 µm before aging and under 0.20 µm after. Between Ra and Rz is a very strong positive Spearman correlation (0.940).

### 2.2. Optical Properties

**Figure 3:**
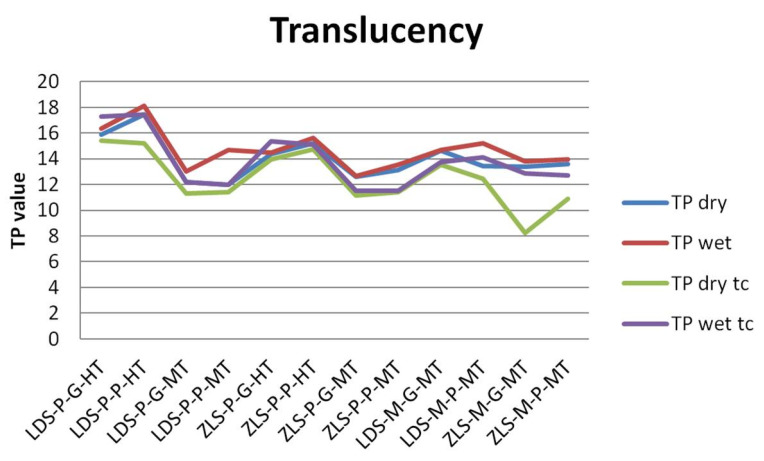
Results of the translucency measurements before and after thermocycling.

**Figure 4:**
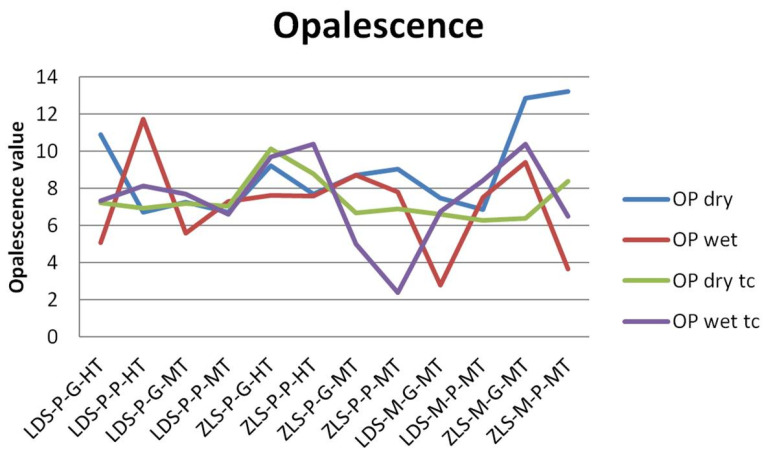
Results of the opalescence measurements before and after thermocycling.

The values of the TP and OP are presented in Figure 3 and Figure 4.

Regarding the TP parameter, LDS samples have higher translucency (mean value = 14.803, and 13.840 after tc), compared to ZLS (mean value = 13.857, and 12.454 after tc), but the difference is insignificant. Between dry and wet samples, the differences are significant after thermal cycling, for wet samples, the TP is higher. Related to processing procedures, the pressed samples and polished are significantly influenced by thermal cycling (*p* = 0.0007, respective *p* = 0.0001), the TP decreases. In all cases, the TP parameter decrease after aging, significant for dry samples. Milled samples have significantly higher TP values than pressed ones, and polished insignificant higher than glazed. Artificial aging decreases significant translucency (Table 2).

Related to the type of processing, TP of MT milled samples is situated between TP of HT and MT pressed samples, both for LDS and ZLS ceramics.

For OP values, the variations are insignificant, within all groups. It can be observed that ZLS samples have higher values than LDS samples, and after thermal cycling opalescence decrease for ZLS. OP values are 7.156 and 7.181 after tc for LDS, 8.797, and 7.633 after tc for ZLS.

A weak positive Spearman correlation was found between TP and OP (0.315).

#### Scanning Electron Microscopy (SEM)

The LDS-P-P specimens, both HT and MT, exhibit typically rod-shaped crystals, forming an interlocking microstructure. The microstructure of LDS-M-P specimens is quite different. Multilayered platelet-shaped crystals embedded in the residual glass matrix formed an interlocking microstructure.

**Figure 5:**
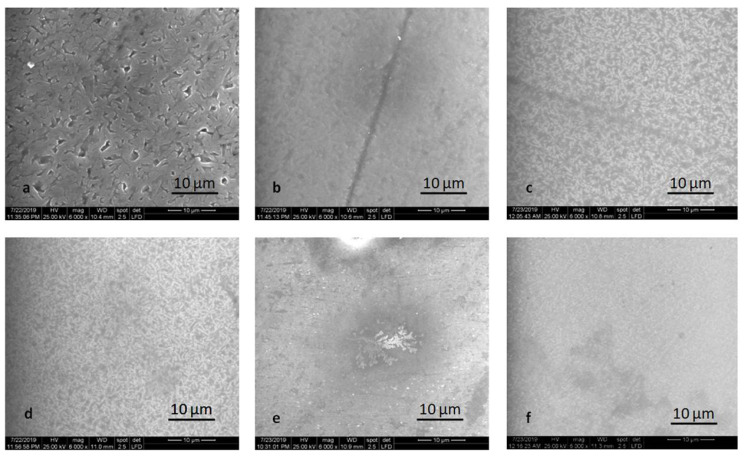
Scanning electron microscopy (SEM) images of the polished samples: (**a**) LDS-P-P-HT, (**b**) LDS-P-P-MT, (**c**) LDS-M-P-MT, (**d**) ZLS-P-P-HT, (**e**) ZLS-P-P-MT, and (**f**) ZLS-M-P-MT.

Another inner structure was observed for the ZLS-P-P and ZLS-M-P specimens. Lath-like crystals of regular and irregular shapes are randomly aligned and embedded in the glass matrix. ZLS is characterized by a homogeneous fine crystalline structure with an average crystal size higher for pressed specimens and higher for HT specimens (Figure 5).

SEM examination revealed noticeable striated patterns on the polished surfaces produced by the instruments.

**Figure 6:**
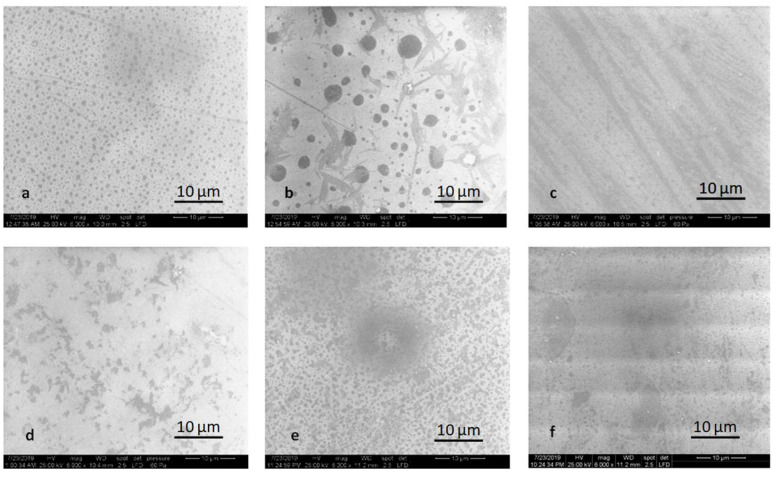
SEM images of the glazed samples: (**a**) LDS-P-G-HT, (**b**) LDS-P-G-MT, (**c**) LDS-M-G-MT, (**d**) ZLS-P-G-HT, (**e**) ZLS-P-G-MT, and (**f**) ZLS-M-G-MT.

Glaze powder and liquid mixture homogeneously dispersed on the surface of the specimen provided smoother surfaces, from SEM point of view, with some inclusions for pressed samples, tiny for HT specimens, and more significant for MT specimens (Figure 6).

**Figure 7:**
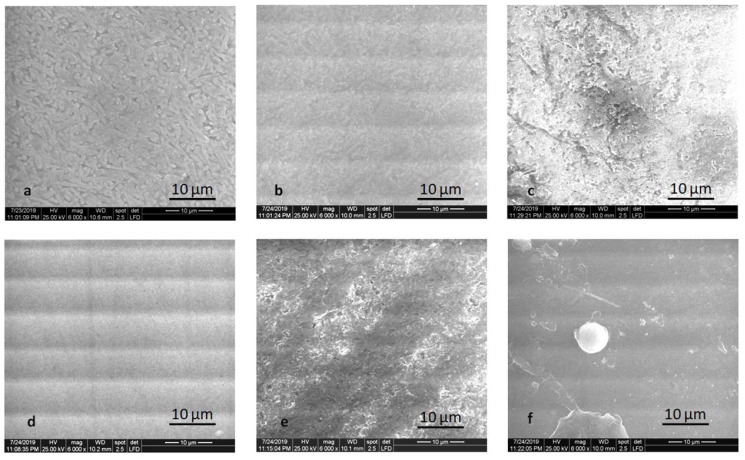
SEM images of the polished samples after thermocycling: (**a**) LDS-P-P-HT, (**b**) LDS-P-P-MT, (**c**) LDS-M-P-MT, (**d**) ZLS-P-P-HT, (**e**) ZLS-P-P-MT, and (**f**) ZLS-M-P-MT.

**Figure 8:**
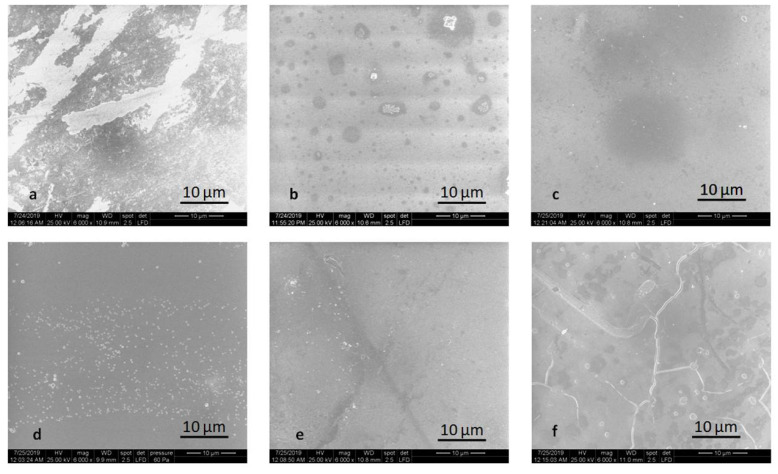
SEM images of the glazed samples after thermocycling: (**a**) LDS-P-P-HT, (**b**) LDS-P-P-MT, (**c**) LDS-M-P-MT, (**d**) ZLS-P-P-HT, (**e**) ZLS-P-P-MT, and (**f**) ZLS-M-P-MT.

The alteration of the surface after thermal cycling is noticeable in Figure 7 and Figure 8. Mainly glazed and milled samples are prone to degradation. The microstructure is no longer visible for the polished LDS-M-P-MT, ZLS-P-P-MT, ZLS-M-P-MT samples. For ZLS-M-P-MT, the crack of the glaze is shown in Figure 8.

### 2.3. Nanosurface Topographic Characterization by AFM

**Figure 9:**
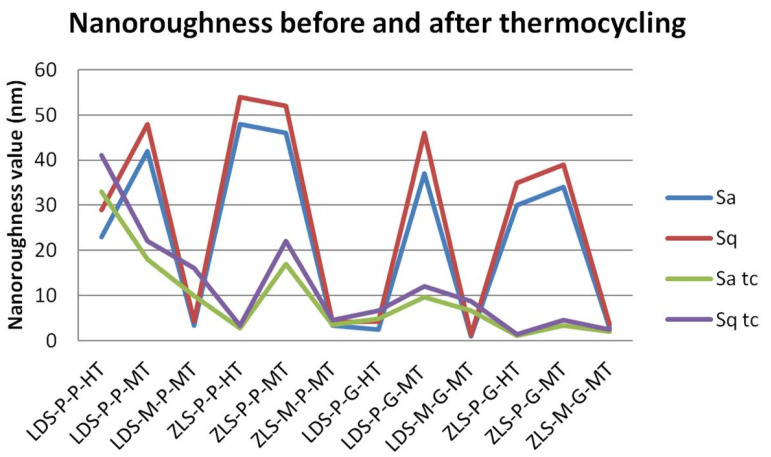
Results of the nanoroughness measurements before and after thermocycling.

**Figure 10:**
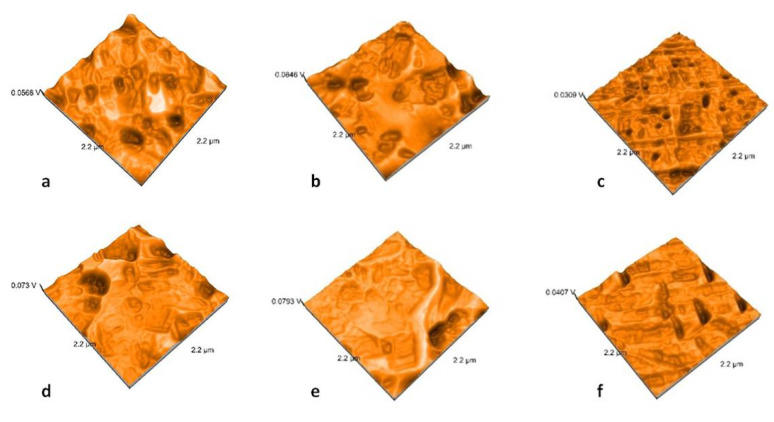
Atomic Force Microscopy (AFM) images of the polished samples: (**a**) LDS-P-P-HT, (**b**) LDS-P-P-MT, (**c**) LDS-M-P-MT, (**d**) ZLS-P-P-HT, (**e**) ZLS-P-P-MT, and (**f**) ZLS-M-P-MT.

**Figure 11:**
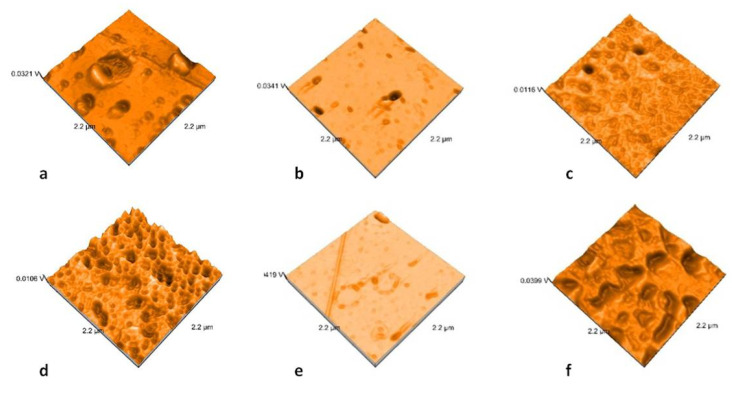
AFM images of the glazed samples: (**a**) LDS-P-G-HT, (**b**) LDS-P-G-MT, (**c**) LDS-M-G-MT, (**d**) ZLS-P-G-HT, (**e**) ZLS-P-G-MT, and (**f**) ZLS-M-G-MT.

**Figure 12:**
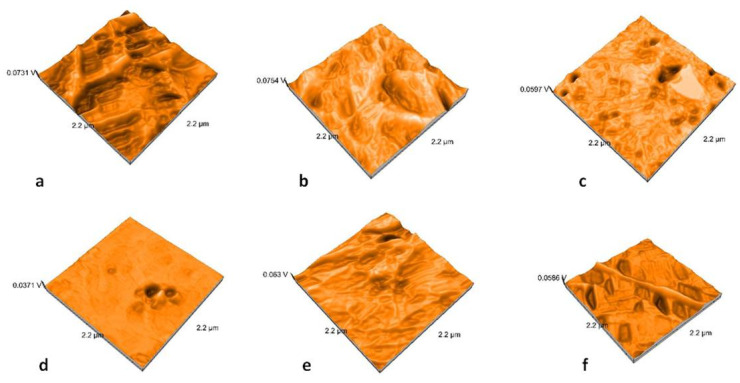
AFM images of the polished samples after thermocycling: (**a**) LDS-P-P-HT, (**b**) LDS-P-P-MT, (**c**) LDS-M-P-MT, (**d**) ZLS-P-P-HT, (**e**) ZLS-P-P-MT, and (**f**) ZLS-M-P-MT.

**Figure 13:**
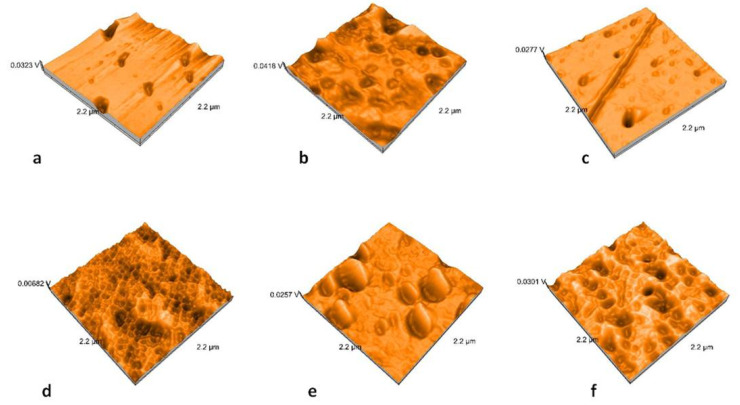
AFM images of the glazed samples after thermocycling: (**a**) LDS-P-G-HT, (**b**) LDS-P-G-MT, (**c**) LDS-M-G-MT, (**d**) ZLS-P-G-HT, (**e**) ZLS-P-G-MT, and (**f**) ZLS-M-G-MT.

Nanoroughness values vary between 0.9 and 48 nm before and between 1.1 and 33 nm after thermal cycling (Figure 9). The decrease in Sa values is significantly influenced by thermal cycling (*p* = 0.0327). Artificial aging generates a more uniform topography of the surfaces (Figure 10, Figure 11, Figure 12 and Figure 13). Sa and Sq values vary in the same direction (very strong positive Spearman correlation 0.989). The mean values of Sa decrease from 22.75 to 9.31 after thermal cycling.

Regarding processing technologies, pressed samples are significantly influenced (*p* = 0.0003) by thermal cycling. There is a significant difference between polished and glazed samples, before thermal cycling (*p* = 0.361); the polished samples have higher nanoroughness than the glazed once.

Related to the type of material, ZLS samples are significantly influenced (*p* = 0.0293) in terms of nanoroughness.

There is a moderate negative Spearman correlation between microroughness Ra and nano roughness Sa (−0.581).

## 3. Discussion

In dentistry, optical properties play an essential role in treatment success and patient satisfaction. Besides color, translucency, and opalescence of restorative materials are critical factors in aesthetic rehabilitation. The very fine microstructure in ZLS could be related to a strong interface between the zirconia enriched matrix and very fine crystals of lithium silicate. The combination of a brittle ceramics matrix enriched with high strength ceramic particles or dispersed oxides can result in a material with relatively good optical properties. The thermal crystallization treatment appeared to be a crucial aspect of the mechanical and optical properties of the material [35].The translucency of dental materials is depended on many factors, such as the ratio of the crystalline/glass phases and the difference in the refractive index between these phases, the morphology of crystals, grain boundaries, pores, second-phase component, additives, and light scattering from the surface. High crystalline content is associated with high opacity when the two phases have different refractive indexes, but the high translucency of LDS glass-ceramics is attributed to the matching of the refractive index of the crystal phase to that of the glass matrix. The opacity of these materials is attributed to the interlocking microstructure of LDS crystals and their sizes [36,37,38].

It was concluded that the difference in TP values might result from the difference in the crystalline content of the materials because an increase in the crystalline content often results in increased opacity. It was reported that the difference in TP values was originated from the different grain size and crystalline structure of the materials.

The materials taken into consideration in this study revealed insignificant differences between TP values for LDS and ZLS glass-ceramics, produced as HT or MT samples. Significant differences are given by the processing technology (pressing or milling, polishing or glazing), for all tested materials. Milled samples have a higher translucency compared to the heat-pressed ceramic samples. For milled MT samples, the translucency is between those of HT and MT pressed samples. Likewise, polished samples have higher translucency. An important practical aspect is related to the recording method. Wetting of the surfaces increases the translucency significantly, and therefore, this must be taken into account because the oral environment is moist.

The artificial aging by thermal cycling reduced the TP values significantly for all samples. The effect of thermal cycling on the optical properties (color and opacity) could be explained with the increase of the crystal size, the orientation of the crystals, and perhaps with the change of the glass matrix [36,37,38,39,40].

The translucency of restorative materials ranges from 9 to 19, at a thickness of 1 mm after other studies can reach 25 [41,42,43]. For the tested samples, the translucency was 12.454–14.803 for 1.5 mm thick samples, both before and after thermal cycling. Values for the translucency for the human structures range between 15 and 19 [44].

Opalescence can improve a dental restoration appearance to look more natural. Based on the results of this study, no parameter does influence the opalescence of the materials, recorded between 7.156 and 8.797. The opalescence parameter for dental structures is between 19.8 and 22.6. Previous studies reported values of 2.5–13.3 for monolithic ceramic restorations [45].

AFM analysis possess an essential advantage in studying ceramic materials because it provides high-resolution and three-dimensional visualization of the studied surface. It showed results that demonstrated that polished surfaces presented significantly higher values when compared to the glazed ones [46,47]. Topographic analyses using AFM bring information at the nano level, and nano roughness is not positively correlated with microroughness. Moreover, in this study, a moderate negative correlation was reported for Ra and Sa parameters (Spearman correlation of −0.581). The microroughness increases and nanoroughness decreases after thermocycling.

Ra values above 0.2 µm have been reported to lead to increased plaque accumulation and periodontal inflammation, as well as a higher risk of dental caries [47,48]. Recorded values for Ra during this study are below this value. The Ra values for glazed samples are significantly higher than for polished, opposite to the Sa values.

Studies found that glazed groups and some polished groups exhibited similar surface roughness. Therefore, the polishing system did not have the same effect on all the ceramic systems [49,50,51]. It was noted the importance of considering the material structure when selecting a polishing method and that leucite content influences polish ability [52,53]. Some authors concluded that glazed lithium disilicate specimens of and zirconia-reinforced lithium silicate possess smoother surfaces compared to the same ceramic polished specimens. Other authors stated that the application of the glaze sprays and glaze paste results in rougher surfaces [54,55,56].

In terms of topography, artificial aging smooths the surfaces, but the difference in microstructure leads to a differential dissolution, with consequences on the microroughness.

The presence of crystalline species within the glass shows the differential dissolution of the glass versus the crystalline phase, resulting in a rougher surface [57]. ZLS samples are significantly influenced in terms of nanoroughness due to the decreased size of the crystal size. The crystal size in ZLS ceramic has been stated to be four–eight-times smaller compared to the LDS ceramics after the crystallization process is finalized [58].

## 4. Materials and Methods 

### 4.1. Sample Preparation

Six monolithic restoration materials were selected: CAD/CAM milled lithium disilicate glass-ceramic (IPS e.max CAD; Ivoclar Vivadent, Schaan, Liechtenstein) MT (medium translucency), hot-pressed lithium disilicate glass-ceramic (IPS e.max Press; Ivoclar Vivadent, Schaan, Liechtenstein) MT and HT(high translucency), CAD/CAM milled zirconia-reinforced lithium silicate ceramic (Vita Suprinity; Vita Zahnfabrik, Bad Säckingen, Germany) MT and hot-pressed zirconia-reinforced lithium silicate ceramic (Celtra Press; Degudent, Hanau, Germany) MT and HT (Table 3).

Round specimens (d = 20.0 mm and h = 1.5 mm) were achieved by heat pressing (Table 4) or cut from partially crystallized blocks.

The crystallization protocol for the ceramic samples respected the manufacturer’s indications, as the samples were fully crystallized in a ceramic furnace (Multimat Touch and Press, Dentsply, Hanau, Germany) at 850 °C for 25 min for IPS e.max CAD, respectively 30 min for Vita Suprinity.

All the specimens were polished to a high-luster finish on all of the surfaces. Further, one side was glazed for each sample, resulting in *n* = 96 surfaces. The samples were divided into 12 groups, according to the material type, processing technology, translucency, polishing, and glazing procedures of the surfaces.

All the specimens were first grounded underwater with a fine-grit diamond-impregnated bur by using a hand-piece and motor. Mechanical polishing was performed by grinding sheets with a grain size of SiC P600–P2000, using a grinding machine (Mecatech 264, Presi, Eybens, France) under running water. The thickness of each specimen was verified using a digital caliper. In this experimental group, one surface of each specimen received two thin layers of specific glaze, according to the manufacturer’s recommendations.

Before investigations, the ceramic specimens were ultrasonically cleaned for about 10 min with distilled water.

### 4.2. Surface Roughness Measurements

Specimens were measured with a contact 2 µm stylus profilometer Surftest SJ-201 (Mitutoyo, Kawasaki, Japan), to evaluate the surface roughness. Arithmetic average roughness (Ra) and maximum absolute vertical roughness (Rz) [32] measurements were performed in 5 different zones, and all data were recorded. The mean value of the five measurements was obtained for each surface. The sampling length was 0.8 mm, and a force of 0.7 mN was applied. All registrations were recorded on dry and wet surfaces (after insertion of the samples in distilled water for 30 min), before and after in vitro aging by thermal cycling. The measured zones of the samples were the same before and after thermocycling.

### 4.3. Optical Properties Measurements

Translucency and opalescence parameters were determined for all specimens before and after thermal cycling. Optical properties were calculated under a D65 illuminant, using a spectrophotometer Vita Easyshade IV (Vita Zahnfabrick, Bad Säckingen, Germany). The spectrophotometer was calibrated before each measurement.

A black (b) and white (w) background were used to assess the measurements, using a grey card (WhiBal G7 (White Balance Pocket Card). L* is a measure of the lightness-darkness of material (perfect black has an L* = 0, and perfect white has an L* = 100). a* coordinate represents the redness (positive value) or the greenness (negative value), while the b* coordinate is a measure of the yellowness (positive value) or the blueness (negative value) [16,36,37].

TP values were calculated using the following equation:

TP = [(L_b_ − L_w_)^2^ + (a_b_ − a_w_)^2^ + (b_b_ − b_w_)^2^]^1/2^ (1)

OP values were calculated using the following equation:

OP = [(a_b_ − a_w_)^2^ + (b_b_ − b_w_)^2^]^1/2^ (2)

### 4.4. Microstructure Analysis by Scanning Electron Microscopy (SEM)

The ceramic samples have been further investigated for microstructure analysis, before and after artificial aging by thermal cycling. Specimens from each group were subjected to an SEM examination (San Francisco Estuary Institute, Richmond, CA, USA). SEM images were made at the accelerating voltage of the electrom beam of 15 kV. Secondary electrons were used to obtain high resolution images. SEM images were used for qualitative analysis to evaluate the relative dimensions and arrangement of the crystals.

### 4.5. Nanosurface Topographic Characterization Using Atomic Force Microscopy (AFM)

Each ceramic sample was examined before and after thermal cycling with an atomic force microscope Nanosurf Easy Scan 2 Advanced Research (NanosurfAG, Liestal, Switzerland), and values for average nanoroughness Sa (nm) and maximal nanoroughness Sq (nm) were registered. The imaging mode is made in contact and the oscillating frequency of the tip is 50 Hz. AFM generated after examination of a three-dimensional image of the sample surface (2.2 µm × 2.2 µm).

### 4.6. In Vitro Artificial Aging Using Thermal Cycling

For artificial aging, a thermocycler (Thermocycler, SD Mechatronik, Feldkirchen-Westerham, Germany) using distilled water with baths of 5 and 55 °C, for 10,000 cycles was used. After initial measurements, the samples were aged, and all registrations were recorded and compared to the initial once.

### 4.7. Statistical Analysis

Statistical inference was performed using the Analyse-it software (Analyse-it Software, Ltd., Leeds, UK). The differences among the variables were made. The unpaired t-test was used to evaluate the comparisons between the means. A *p*-value of under 0.05 was considered statistically significant. Spearman correlation was used to assesses monotonic similar or dissimilar relationships (whether linear or not) between variables. It measures the strength of association between variables and the direction of the relationship. The significance was related to 00–0.19 “very weak”, 0.20–0.39 “weak”, 0.40–0.59 “moderate”, 0.60–0.79 “strong”, and 0.80–1.0 “very strong”.

## 5. Conclusions

Within the limitations of this study, the following conclusions can be drawn.

1. The differences recorded between translucency and opalescence of ZLS and LDS ceramics are not significant. Regarding processing technologies, milling and polishing are associated with significantly higher translucencies.
2. Thermal cycling has a significant influence on the translucency of ZLS and LDS glass-ceramic materials. The most harmful effect is on the pressed and polished samples, the consequences being the decrease in translucency.
3. The surface roughness values of all samples are under an acceptable limit of 0.2 µm. Among the processing procedures, glazing increases the microroughness significantly, and milling leads only to an insignificant increase.
4. In terms of nanoroughness, ZLS samples are significantly influenced due to the size of the crystals.
5. A negative correlation was recorded for micro- and nano roughness parameters. Ra and Sa’s values are significantly influenced by in vitro aging.

## Figures and Tables

**Table 1:** *p*-values for the roughness variations of the tested samples.

Type of Tested Samples	*p*-Value
dry-wet	0.1157
ZLS-LDS	0.7228
G-P	<0.0001
P-M	0.1376
dry tc-wet tc	0.0005
ZLS tc-LDS tc	0.2344
G tc-P tc	0.0018
P tc-M tc	0.0593
all-all tc	<0.0001

**Table 2:** *p*-values for the translucency variations of the tested samples.

Tye of Tested Samples	*p*-Value
dry-wet	0.0102
ZLS-LDS	0.0117
G-P	0.0228
P-M	0.0030
dry tc-wet tc	0.0035
ZLS tc-LDS tc	0.0065
G tc-P tc	0.4460
P tc-M tc	0.2511
all-all tc	<0.0001

**Table 3:** Materials involved in the study.

Restoration Material	Manufacturer	Class of Ceramic	Processing	Composition	Translucency/Shade
IPS e.max CAD	Ivoclar Vivadent, Schaan, Liechtenstein	lithium disilicate glass-ceramic (LDS)	CAD/CAM milled	70 vol% of the crystalline phase (average particle size 0.2–1.0 µm) incorporated in a glassy matrix	MT/A2
IPS e.max Press	Ivoclar Vivadent, Schaan, Liechtenstein	lithium disilicate glass-ceramic (LDS)	hot-pressed	lithium disilicate crystals (approx. 70%), Li_2_Si_2_O_5_ crystals (3–6 µm length)	MT/A2
					HT/A2
Vita Suprinity	Vita Zahnfabrik, Bad Säckingen, Germany	zirconia-reinforced lithium silicate ceramic (ZLS)	CAD/CAM milled	silica (55–65 wt.%), lithia (15–21 wt.%), zirconia (8–12 wt.%) particle size 0.5–0.7 µm	MT/A2
Celtra Press	Degudent, Hanau, Germany	zirconia-reinforced lithium silicate ceramic (ZLS)	hot-pressed	a glass matrix and lithium disilicate crystals 1.5 µm plus nanoscale lithium phosphate, 10% zirconia (ZrO_2_)	MT/A2
					HT/A2

**Table 4:** Parameters for ceramics pressing.

Pressing Parameters for Hot-Pressed Ceramics	Starting Temperature	Hold Time	Vacuum Level	Press Time	Heat Rate	Press Temperature	Press Pressure
IPS e.max Press	700 °C	29 min	47 hPa	1 min	60 °C/min	915 °C	3 bar
Celtra Press	700 °C	30 min	45 hPa	3 min	40 °C/min	860 °C	3 bar
